# Supplementary material for: Coexistence or conflict: Black bear habitat use along an urban-wildland gradient
Source: PLoS One. 2022 Nov 29;17(11):e0276448. doi: 10.1371/journal.pone.0276448 (PMC9707782; doi:10.1371/journal.pone.0276448)
Supplement: S1 Table — Predictor variables used to model black bear habitat use in Sooke, Vancouver Island, Canada, between 2018–2019. Variables all derived within 150 m radius buffers around camera trap locations unless otherwise noted. Weighted buffers reduce the contribution of raster layer cells not fully within the circular buffer by the percent excluded. (DOCX) [file pone.0276448.s001.docx]

Table S1: Predictor variables used to model black bear habitat use in Sooke, Vancouver Island, Canada, between 2018-2019. Variables all derived within 150 m radius buffers around camera trap locations unless otherwise noted. Weighted buffers reduce the contribution of raster layer cells not fully within the circular buffer by the percent excluded.

| Predictor Variable | Source |  | Description |
| --- | --- | --- | --- |
| Human Density | Gridded Population of the World |  | Average number of people per square kilometre within weighted buffer |
| Road Density | BC Digital Road Atlas |  | Total length of roads in km / km^2^ of buffer area |
| Trail Density | CRD Regional Parks |  | Total length of trails in km / km^2^ of buffer area |
| Elevation | ASTER Global Digital Elevation |  | Average height above sea level within weighted buffer |
| Distance-to-Agriculture | CRD Regional Parks |  | Average distance from camera location to agriculture land cover cells within weighted buffer |
| Distance-to-Urban | CRD Regional Parks |  | Average distance from camera location to urban land cover cells within weighted buffer |
| Enhanced Vegetation Index (EVI) | MODIS’s 250m 16-day layers (MOD13Q1) |  | Average based on number of days the 16-day MODIS window had within calendar month within weighted buffer |
| Distance-to- Freshwater | CRD Regional Parks |  | Distance from camera location to closest freshwater resource (lakes, ponds, rivers, creeks) |
| Presence/absence of Salmon | Charters Creek Hatchery, *unpublished data* |  | Binary variable, known presence or absence of salmon in rivers and creeks within buffer of camera site per calendar month |
| # Reported Conflicts | British Columbia Conservation Officer Service, Human-Wildlife Conflict Reporting Database |  | Number of reported conflicts within 500 m buffer of camera site within study year |
