# Supplementary material for: Coexistence or conflict: Black bear habitat use along an urban-wildland gradient
Source: PLoS One. 2022 Nov 29;17(11):e0276448. doi: 10.1371/journal.pone.0276448 (PMC9707782; doi:10.1371/journal.pone.0276448)
Supplement: S2 Table — Mean values of predictor variables by strata used to model black bear habitat use in Sooke, Vancouver Island, Canada, between 2018–2019. (DOCX) [file pone.0276448.s002.docx]

Table S2: Mean values of predictor variables by strata used to model black bear habitat use in Sooke, Vancouver Island, Canada, between 2018-2019.

| Variable | Wild | Rural | Urban |
| --- | --- | --- | --- |
| Human Density | 85.214 | 158.145 | 650.156 |
| Road Density | 3.641 | 4.384 | 9.051 |
| Trail Density | 6.765 | 0.925 | 0.341 |
| Elevation | 159.585 | 49.115 | 57.887 |
| Distance-to-Agriculture | 2523.105 | 742.942 | 511.435 |
| Distance-to-Urban | 141.089 | 92.839 | 18.047 |
| Enhanced Vegetation Index (EVI) | 3742.518 | 3684.320 | 3456.462 |
| Distance-to-Freshwater | 234.551 | 403.494 | 157.279 |
| Presence/absence of Salmon | 0.064 | 0.057 | 0.014 |
| # Reported Conflicts | 0 | 0.101 | 0.203 |
