# Supplementary material for: Coexistence or conflict: Black bear habitat use along an urban-wildland gradient
Source: PLoS One. 2022 Nov 29;17(11):e0276448. doi: 10.1371/journal.pone.0276448 (PMC9707782; doi:10.1371/journal.pone.0276448)
Supplement: S3 Table — All candidate for black bear habitat use as measured by monthly camera trap detection rates, from 54 camera traps sampled in and around Sooke, BC, Canada from July 2018 –July 2019 using zero-inflated GLMMs. Evaluated predictor variables extracted from a 150m buffer around camera locations include HD = human density, RD = road density, EVI = enhanced vegetation index, DUrb = distance-to-urban, DAg = distance-to-agriculture, Con = conflict (500 m buffer), DW = distance-to-freshwater, Sal = salmon, Ele = elevation, and TD = trail density. All models also have site as a random effect and number of active days as an offset. Df is the degrees of freedom of the model, within ΔAICc is the difference in AICc scores from the top model within a set, between ΔAICc is the difference in top models between sets, Akaike weight is the relative likelihood of a model divided by the sum of those values across all models. (DOCX) [file pone.0276448.s003.docx]

Table S3: All candidate models for black bear habitat use as measured by monthly camera trap detection rates, from 54 camera traps sampled in and around Sooke, BC, Canada from July 2018 – July 2019 using zero-inflated GLMMs. Evaluated predictor variables extracted from a 150m buffer around camera locations include HD = human density, RD = road density, EVI = enhanced vegetation index, DUrb = distance-to-urban, DAg = distance-to-agriculture, Con = conflict (500 m buffer), DW = distance-to-freshwater, Sal = salmon, Ele = elevation, and TD = trail density. All models also have site as a random effect and number of active days as an offset. Df is the degrees of freedom of the model, within ΔAICc is the difference in AICc scores from the top model within a set, between ΔAICc is the difference in top models between sets, Akaike weight is the relative likelihood of a model divided by the sum of those values across all models.

| Hypothesis | Predictor Variables | df | Within  ΔAICc | Between  ΔAICc | Akaike  Weight |
| --- | --- | --- | --- | --- | --- |
| Conflict  (EVI) | HD + HD^2^ + RD + EVI + DAg + DUrb + Ele + TD + TD*HD  HD + HD^2^ + RD + EVI + DAg + DUrb + Ele + TD  HD + RD + EVI + DAg + DUrb + Ele + TD + TD*HD  HD + RD + EVI + DAg + DUrb + Ele + TD | 13  12  12  11 | 0.0  0.6  2.2  2.2 | 0.0 | 0.41  0.31  0.14  0.14 |
| Conflict (distance-to-forest) | HD + HD^2^ + RD + DAg + DUrb + DFP + DFI + Ele + TD + TD*HD  HD + HD^2^ + RD + DAg + DUrb + DFP + DFI + Ele + TD  HD + RD + DAg + DUrb + DFP + DFI + Ele + TD + TD*HD  HD + RD + DAg + DUrb + DFP + DFI + Ele + TD | 14  13  13  12 | 0.0  1.4  3.6  4.1 | 6.1 | 0.562  0.274  0.093  0.071 |
| Full Model | HD + HD^2^ + RD + EVI + DAg + DUrb + Con + DW + Sal + Ele + TD + TD*HD  HD + HD^2^ + RD + EVI + DAg + DUrb + Con + DW + Sal + Ele + TD  HD + RD + EVI + DAg + DUrb + Con + DW + Sal + Ele + TD + TD*HD  HD + RD + EVI + DAg + DUrb + Con + DW + Sal + Ele + TD | 16  15  15  14 | 0.0  0.6  2.5  2.6 | 2.7 | 0.44  0.32  0.13  0.12 |
| Anthropogenic | HD + HD^2^ + RD + DAg + DUrb + Con + TD + TD*HD  HD + HD^2^ + RD + DAg + DUrb + Con + TD  HD + RD + DAg + DUrb + Con + TD + TD*HD  HD + RD + DAg + DUrb + Con + TD | 12  11  11  10 | 0.0  0.7  3.4  3.4 | 4.7 | 0.483  0.340  0.089  0.087 |
| Environmental | EVI + DW + Sal + Ele | 8 | NA | 11.5 | NA |
| Null |  | 4 | NA | 24.1 | NA |
