# Supplementary material for: Coexistence or conflict: Black bear habitat use along an urban-wildland gradient
Source: PLoS One. 2022 Nov 29;17(11):e0276448. doi: 10.1371/journal.pone.0276448 (PMC9707782; doi:10.1371/journal.pone.0276448)
Supplement: S4 Table — Number of independent black bear detections in the day versus night at urban, rural, and wild camera trap sites (n = 548). (DOCX) [file pone.0276448.s004.docx]

Table S4: Number of independent black bear detections in the day versus night at urban, rural, and wild camera trap sites (n=548).

|  | **Day** | **Night** |
| --- | --- | --- |
| **Urban** | 37 | 40 |
| **Rural** | 195 | 173 |
| **Wild** | 79 | 24 |
