# Supplementary figures and images for: Coexistence or conflict: Black bear habitat use along an urban-wildland gradient
Source: PLoS One. 2022 Nov 29;17(11):e0276448. doi: 10.1371/journal.pone.0276448 (PMC9707782; doi:10.1371/journal.pone.0276448)

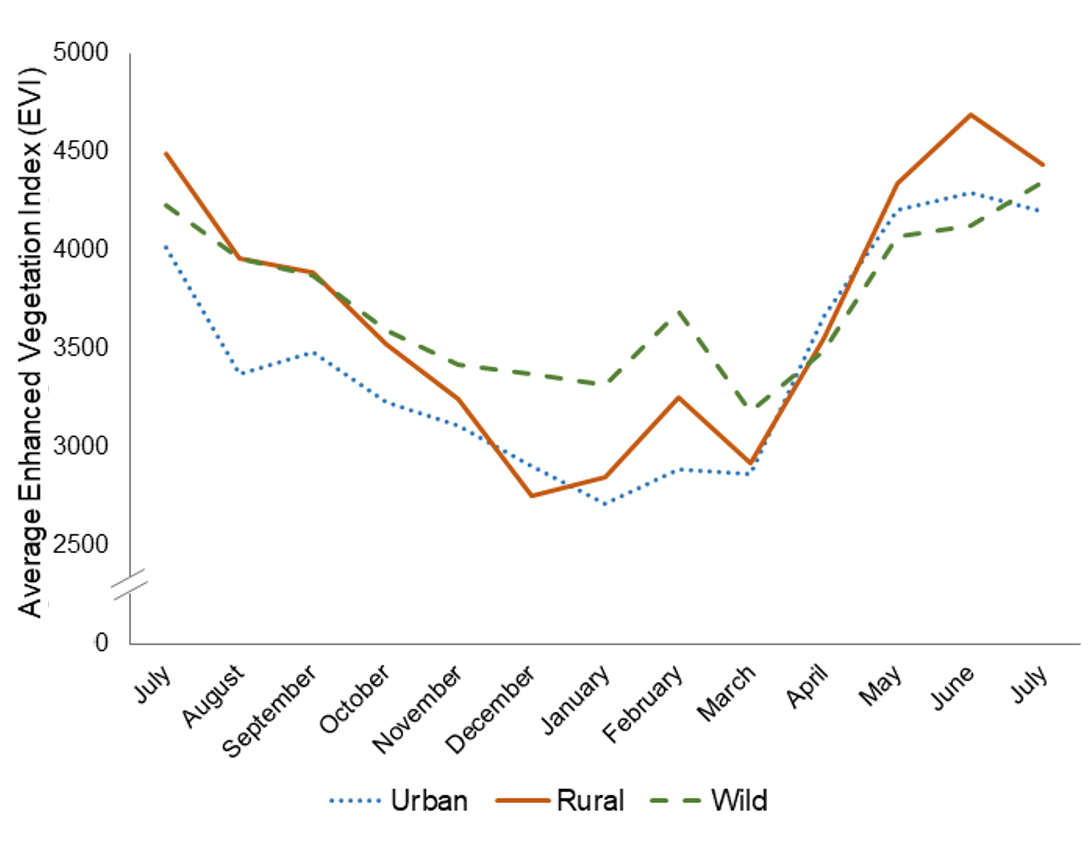

Supplement: S1 Fig — Enhanced Vegetation Index (EVI) averaged within sampling strata (urban, rural, or wild) across 54 camera-trap sites in Sooke, BC, Canada sampled from July 2018–2019. (TIF) [file pone.0276448.s005.tif]
