# Supplementary material for: Coexistence or conflict: Black bear habitat use along an urban-wildland gradient
Source: PLoS One. 2022 Nov 29;17(11):e0276448. doi: 10.1371/journal.pone.0276448 (PMC9707782; doi:10.1371/journal.pone.0276448)
Supplement: Attachment — Submitted filename: PONE-D-22-12898_review.pdf [file pone.0276448.s006.pdf]

Review for:

Coexistence or conflict: black bear habitat use along an urban-wildland gradient

Note: This was written in markdown format. I've also included a PDF if that is easier for you to read.

In this paper the authors look at spatio-temporal variation in the distribution of black bears in an area where conflict between black bears and humans is high. Additionally, they also look at the correlation between black bear detections and the probability of black-bear conflict that was estimated from previous research. In general, I think this is an interesting and well written piece of research. I often found myself in agreement with the logical flow of the research, the modeling choices made, and the interpretation of said models. Great work!

Perhaps my biggest concern is the secondary modeling approach, which treats the estimated conflict probability as known, when in fact it is something that is estimated with error. In my review below I have a number of hopefully helpful citations about this specific issue for the authors.

I also suggest the authors look at a recent article of mine (Fidino et al. 2022), which shows how integrated models could be use to combine human-wildlife conflict data with camera trap data. I'm not on the hunt for a citation here, but since it is relevant to this piece of research I am sharing in case you were interested in how to do this in the future.

```
Fidino, M., Lehrer, E. W., Kay, C. A., Yarmey, N. T., Murray, M. H., Fake, K., ... &
Magle, S. B. (2022). Integrated species distribution models reveal spatiotemporal
patterns of human–wildlife conflict. Ecological Applications, e2647.
```

If you have any specific questions about any of my comments, feel free to email me at mfidino@lpzoo.org. Again, great work on this!

Abstract

Line by line comments

Line 46: The 'such as' breaks up this sentence in a weird way. I think you can remove it and the examples become a little more clear.

Introduction

Top-level thoughts

1. Well written introduction, I only have some minor comments on some of the wording / sentence structure.

Line by line comments

Line 64: Is sustainable the correct word here? I think it could be, but also it tends to have a bit of a positive connotation to it, so it reads a little off to say sustainable risks of death. Maybe 'acceptable' could be used instead? Just a minor point, feel free to ignore if you disagree.

Line 69: This sentence is a little circular. Changes to avoid direct conflict can increase direct conflict when risk of direct conflict is lower. I think it's the second use of direct conflict, which makes it feel a little redundant. Could that bit right after the first (e.g.,) just get removed? I think the point still comes across.

Line 76-77: Is 'year round' needed when you already state they are available consistently? "...as they are predictable, consistent, and spatially aggregated sources of..."

Line 82-84: Does it help here to remind the reader that the urban-wildlife interface is increases, and therefore increased risk of ecological traps for such species?

Line 88: ...to signal wildlife mortality risks...

Line 98: ...and can alter their foraging patterns to...

Line 103: ..., and can result in seasonal...

Line 103-105: Sentence starting with "If such attractants" reads a little weird. Maybe it's the 'attractants can attract animals' part or that the '...and food-conditioning' bit at the end feels like a different thought tacked on.

Line 105 - 108: This sentence could be made more active (and therefore a little more succinct). "In Nevada, urban black bears are less active each day (as they satisfied their caloric needs faster), more nocturnal, and hibernate less."

Line 116-120: Conflict probability of what? Do you mean human-wildlife conflict probability in general? Human-bear conflict in particular?

Line 123-125: Great point, and the introduction sets a really strong foundation for the need to study this.

Methods

Top-level thoughts

1. I would imagine that the distance between camera trapping locations would be species specific to maintain spatial independence. For black bear, multiple camera traps can be contained within the home range of a single bear, and therefore I would assume that spatial independence is not met. This citation is also a pre-print, though it's been around for 12 years. Maybe just soften the language here (e.g,. say to increase spatial independence rather than maintain spatial independence).

2. Was the sampling effort term included as an offset to the model or did you just include it as a covariate? If the latter was done, I would encourage adding a log offset term instead, as that is a more standard approach when there is variation in sampling effort. If I had to guess, you used a log-offset, so just be specific about that here in the methods.

3. I could see some readers get their hackles up about not using an occupancy model here. I leave it to the authors, but it may help to just get ahead of it here (by using a zero-inflated model you may be estimating habitat use conditional on presence anyways, depending on the class of zero-inflated model you fit). Likewise, occupancy models are literally just zero-inflated logistic regression, so your approach has substantial overlap. For example, the old

MacKenzie et al. occupancy modeling book talks about these similarities on about page 135 (i.e., the use of the zero-inflated binomial to model occupancy).

```
MacKenzie, D. I., Nichols, J. D., Royle, J. A., Pollock, K. H., Bailey, L. L., &
Hines, J. E. (2017). Occupancy estimation and modeling: inferring patterns and
dynamics of species occurrence. Elsevier.
```

4. There are a few different kinds of zero-inflated glmms, can you be a little more specific? For example, what is the error distribution that was used (negative binomial, Poisson, etc.). Is this a two part model and therefore the conditional model cannot include zeroes or is it a mixture model and therefore the conditional models can include zero? A little more explanation here would be helpful. note: I see now that the distributional information is shared much lower, around line 297. I'd move that little bit of info upwards so it's not separated from when you introduce the modeling framework.

5. Thank you for using a set of candidate models to assess your different hypotheses. Solid approach. Given the two set hypotheses brought up in the introduction, in may help to add a little more connection between those hypotheses and the set of candidate models given that there are two hypotheses but four candidate models. You should also fit a fifth null model as well (just the active days term plus the site random effect). Given the results I suspect the null will provide the worst fit (highest delta AIC), but it's nice to demonstrate this.

6. Based on the introduction (lines 133-134) I thought that modeled conflict probabilities would be incorporated into your glmms, but instead it looks like counts of conflict are included instead. I suspect other readers will also be confused about this. What makes this more confusing, is that there is a second batch of models done that uses the estimated conflict probabilities.

7. Why conflicts over the year instead of conflicts per month?

8. Using the output from one model as a predictor in another is okay, but the uncertainty of those estimates should also be propagated into the secondary model. From my reading of this secondary model set, I'm guessing that these spatially explicit probabilities are treated as known (i.e., measured without error). If this is the case, using such predictions in secondary analysis leads to anticonservative tests because this error is excluded from further tests (i.e., estimates are too precise). Some papers about this topic that the authors may find useful include:

```
Hadfield, J. D., Wilson, A. J., Garant, D., Sheldon, B. C., & Kruuk, L. E. (2010). The
misuse of BLUP in ecology and evolution. The American Naturalist, 175(1), 116-125.

Houslay, T. M., & Wilson, A. J. (2017). Avoiding the misuse of BLUP in behavioural
ecology. Behavioral Ecology, 28(4), 948-952.

Link, W. A. (1999). Modeling pattern in collections of parameters. The Journal of
wildlife management, 1017-1027.
```

The Houslay & Wilson paper is open access, so that is where I'd start. I've personally found this easiest to account for in a Bayesian framework (e.g., if you have the mean and SE of each prediction you can set a prior for each data point to propagate that uncertainty), but there are likely ways to deal with this in a frequentist framework as well (e.g., bootstrapping, but resampling the predicted covariate instead of the response variable).

9. What is a reported conflict in these data? Do they vary in severity?

Results

Top-level thoughts

1. Failing to detect an effect does not mean that there was no effect (e.g., line 391 - 392). I'd just reword to "We failed to detect an effect of road density" so that you avoid confirming the null (which these tests do not do). Other than that, great breakdown of the results.

Discussion

Top-level thoughts

1. Given the conflicting hypotheses, do the authors feel that one hypothesis was supported more than the other?

2. Any caveats worth bringing up here? For example, there was the assumption that EVI indicates forage availability. Is it possible for there to be human-bear conflicts that go unreported and so the conflicts / year metric used may have some error?

Line by line comments

Line 530: You used ecological trap earlier.

Tables & figures

Top-level thoughts

1. The axis text on many of the figures is a very light gray, I'd suggest replacing with black to make it easier to read.

2. You could increase the line width for the 95% CI's on figure 3&4, plus the mean estimate on figure 5.
